# Early Short-Term Postoperative Mechanical Failures of Current Ceramic-on-Ceramic Bearing Total Hip Arthroplasties

**DOI:** 10.3390/ma13235318

**Published:** 2020-11-24

**Authors:** Mariano Fernández-Fairén, Ana Torres-Perez, Roman Perez, Miquel Punset, Meritxell Molmeneu, Monica Ortiz-Hernández, José María Manero, Javier Gil

**Affiliations:** 1 Bioengineering Institute of Technology, Facultat de Medicina y Ciencias de la Salud, Universitat Internacional de Catalunya, 080195 Barcelona, Spain; mferfai@gmail.com (M.F.-F.); rperezan@uic.es (R.P.); 2 Hospital Universitario Santa Lucía, Calle Mezquita, s/n, 30202 Cartagena, Spain; anatpz@gmail.com; 3 Biomaterials, Biomechanics and Tissue Engineering Group (BBT), Department of Materials Science and Engineering, Escola d’Enginyeria de Barcelona Est (EEBE), Universitat Politècnica de Catalunya (UPC), Carrer de Jordi Girona 1, 08034 Barcelona, Spain; miquel.punset@upc.edu (M.P.); meritxell.molmeneu@upc.edu (M.M.); monica.ortiz-hernandez@upc.edu (M.O.-H.); jose.maria.manero@upc.edu (J.M.M.); 4 Barcelona Research Centre in Multiscale Science and Engineering, Technical University of Catalonia (UPC), Av. Eduard Maristany, 10–14, 08019 Barcelona, Spain; 5 UPC Innovation and Technology Center (CIT-UPC), Technical University of Catalonia (UPC), C. Jordi Girona 3–1, 08034 Barcelona, Spain

**Keywords:** ceramic-on-ceramic, total hip replacement (THR), catastrophic fracture, acetabular cup, chipping, yttria-stabilized tetragonal zirconia (Y-TZP), fractographic analysis, squeaking, fatigue, postoperative fracture

## Abstract

Although ceramic-on-ceramic (CoC) bearings have been shown to produce the smallest amount of wear volume in vitro as well as in vivo studies when used for total hip arthroplasties (THA), concerns about the failure of these bearing surfaces persist due to early failures observed after short postoperative time. In this study, an exhaustive analysis of the early failure occurred on the new generation of ceramic bearings, consisting of a composite alumina matrix-based material reinforced with yttria-stabilized tetragonal zirconia (Y-TZP) particles, chromium dioxide, and strontium crystals, was performed. For this study, 118 CoC bearings from 117 patients were revised. This article describes a group of mechanical failure CoC-bearing BIOLOX THA hip prosthesis patients without trauma history. The retrieved samples were observed under scanning electron microscopy (SEM), composition was analyzed with energy dispersive X-ray spectroscopy (EDX), and damaged surfaces were analyzed by grazing-incidence X-ray diffraction (GI-XRD) and white light interferometry. In the short term, CoC articulations provided similar mechanical behavior and functional outcome to those in XLPE cases. However, 5% more early mechanical failures cases were observed for the ceramic components. Although the fracture rate of third generation CoC couples is low, the present study shows the need to further improve the third generation of CoC-bearing couples for THA. Despite the improved wear compared to other materials, stress concentrators are sources of initial crack propagation, such as those found in the bore-trunnion areas. Moreover, in view of the evidence observed in this study, the chipping observed was due to the presence of monoclinic phase of the Y-TZP instead of tetragonal, which presents better mechanical properties. The results showed that total safety after receiving a THA is still a goal to be pursued.

## 1. Introduction

According to the European population projections (EUROSTAT), the median age of the population has been continuously increasing in all EU countries during the last 20 years [1]. If the improvements in life expectancy continue in the future, the share of the EU elderly (65+) population will grow from 18.9% to 28.5%, while the share of the Europe elderly (80+) population will grow from 5.3% to 11.1%, between 2014 and 2050 [1]. The progressive life expectancy increase and its consequent rise of aging associated degenerative diseases, together with the fact that hip arthroplasty has been extended to younger and more active cohorts, represent an urgent need to develop new long lasting orthopedic prostheses [2,3,4].

Total hip replacement (THR) is one of the most applied surgical procedures with satisfactory results [5,6]. Metal-on-polyethylene (MOP) combination has been the most commonly implanted bearing surface for decades since it was taken as the THR gold standard [7,8]. However, polyethylene debris can induce periprosthetic inflammation and may produce osteolysis and subsequent implant failure [9,10,11,12,13]. Metal-on-metal (MOM) prostheses, which are less frequently used, present lower values of wear but higher level of metals ions associated with possible local and systemic adverse tissular effects [14,15,16,17]. On the other hand, ceramic-on-ceramic (CoC) bearings are manufactured with fully oxidized materials that are biologically inert and do not produce the same level of inflammatory response as polyethylene or metal when wear particles are released [18,19]. This outstanding tribological properties of CoC bearings made them a better election as an ideal implant for younger patients undergoing THR [9,10,11,12,20,21,22,23,24,25].

CoC bearings have shown the smallest wear volume both in vitro and in vivo studies [25,26,27,28]. In addition, ceramic materials present higher hardness and scratching resistance [18]; better lubrication properties as a result of their hydrophilic character [29]; and lower friction coefficient, resulting in smoother surface and improved quality. Furthermore, several studies demonstrated that CoC bearings significantly decreased the risks of osteolysis, aseptic loosening, and revision in comparison to MOP [30,31].

However, many concerns are related to the use of CoC (THA), such as groin pain, noise, bearing fractures, squeaking, aseptic loosening, and trunnionitis, among others [32,33]. The main problem related to ceramic materials is their brittleness, as well as fracture facility, which remains one of the most important complications to be considered [31]. According to some authors, intraoperative ceramic fracture can occur during the process of inserting a ceramic liner into a metal acetabular cup [34]. Additionally, postoperative ceramic fracture may be multifactorial and can occur as a result of instability trauma and even during normal functional conditions [35,36,37,38]. Instability and impingement of the components may be also pejorative factors to consider. Other studies point out the use of small femoral heads and short-neck heads as a cause for CoC fractures [35]. The heterogeneity of the material and design facts can act as stress risers leading to the propagation of cracks and increasing the risk of failure [38,39,40,41].

The BIOLOX THA, a first generation of alumina bearings, were first introduced in 1974. Subsequent second and third generations were introduced in 1992 and 1995 (BIOLOX-Forte), respectively [42,43]. The appearance of new technologies and manufacturing industries has enabled continuous improvement to overcome the drawbacks of early generations, leading to a fourth generation of CoC bearings that incorporates yttria-stabilized tetragonal zirconia (Y-TZP) into alumina matrix. This new generation is marketed as Biolox Delta ceramic bearings and was introduced by CeramTec AG (Plochingen, Germany) in 2004 [43]. The aim of this composite is to reduce both the risk of fracture and wear rate, as well as to obtain excellent scratch resistance together with low coefficient of friction [44]. This new ceramic consists of 82% alumina, 17% zirconia, and 0.5% chromium oxide to improve hardness and wear characteristics, and 0.5% strontium crystals to diffuse crack energy and help limit crack propagation [34,45,46]. In order to obtain a durable fixation of the socket, the ceramic insert is placed into a Ti6Al4V shell press-fitted into the bone, and provided with screws for fixation [39,41,47,48,49,50,51,52,53,54].

The aim of this study is to perform a retrospective evaluation, if necessary, after primary total hip arthroplasty and determine the causes of early loosening of some acetabular cups and some postoperative fractures. The null hypothesis was that there were no problems of fracture related to the prosthesis design and/or the material.

## 2. Materials and Methods

**Figure 1:**
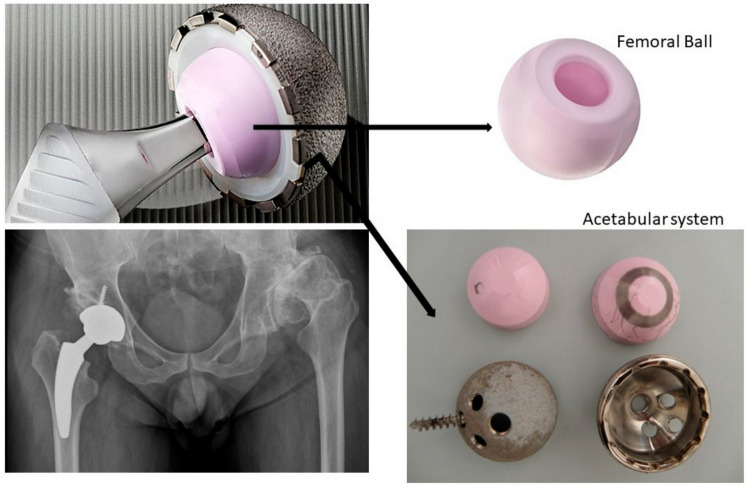
Scheme of the total hip arthroplasty studied.

A total of 117 patients (118 hips) received a cementless CoC Biolox delta THA (CeramTec AG, Plochingen, Germany), distributed in 47 men (47 hips) and 70 women (71 hips) (see Supplementary Materials). Figure 1 shows a scheme of the total hip arthroplasty studied. The average ages at the time of surgery were 45 and 43 years old for men and women, respectively, ranging from 22 to 69 years old for the whole cohort. All patients completed clinical and X-ray revisions after 2 years of follow-up. The implants analyzed in this study were collected after mechanical failure between 2015 and 2018. The study was approved by the Tres Torres Hospital Ethics Committee (reference number 42-2014) at the date of 4 February 2014 in Barcelona (Spain). The cases included in this study were preoperatively diagnosed with osteoarthritis (40%), hip dysplasia development (21%), rheumatoid arthritis (10%), avascular necrosis (10%), juvenile rheumatoid arthritis (9%), and others (10%). The exclusion criteria were previous total hip replacement, previous hemi-arthroplasty, and fusion on the ipsilateral side.

Three surgeons performed all procedures through a direct lateral (77%) or posterior approach (23%). Head size was determined by the inner diameter of the ceramic liner for the corresponding cup size. Two different combinations of THA were assessed: 107 patients received uncemented TRILOGY acetabular cups (Zimmer, Irvine, CA, USA) with uncemented FITMORE femoral stems (Zimmer), and 11 cases received TRINITY acetabular cups (Corin, Cirencester, UK) with MINIHIP femoral stems (Corin, Cirencester, UK). The head diameter of the analyzed acetabular cups ranged from 28 to 32 mm. In all cases, the acetabular fixation was assured with screws.

All patients were evaluated clinically and by X-ray at 6 weeks, 3 months, and 1 year post-surgery. In each follow-up, serial anteroposterior radiographs of the pelvis, and anteroposterior and lateral radiographs of the operated pelvis were taken.

Six THAs with ceramic components were retrieved following the The ISO 12891- 2015 “Retrieval and analysis of surgical implants—Part 1: Retrieval and handling” and were carefully analyzed to determine the cause of their mechanical failure [54]:

1. A total of 4 out of the 6 showed chipping of the liner.
  - One of these cases also presented fracture of the liner head. It corresponded to a 25-year-old woman suffering from dysplasic hip. She received an uncemented Trilogy acetabular cup (Zimmer, USA) with an uncemented Fitmore femoral stem (Zimmer, Warsaw, IN, USA), using a 28 mm diameter head in a 48 mm diameter liner and short neck.
  - In two other cases of liner chipping, the acetabulum was vertically oriented and retroverted. One of them experienced dislocation and closed reduction in the immediate postoperative.
  - The component orientation was judged radiologically perfect in the fourth case, and there was no trauma or instability.
2. The remaining two cases operated using a Trinity cup (Corin, Cirencester, UK) and a MiniHip stem (Corin, Cirencester, UK). Both components were revised for disassembly of the ceramic insert from the metallic cup caused by the protruding head of fixation screws.

The patients’ personal data were masked off from the analyzed components the corresponding X-ray images, and thus the observational retrospectives studies were carried out using existing information that did not contain personal data and did not require approval by the Ethical Committee or Inspection Committee in our academic system.

The retrieved samples were observed under scanning electron microscopy (SEM) at 10 kV using a Neon 40 Focused Ion Beam Scanning Electron (FIB-SEM) microscope (Carl Zeiss NTS GmbH, Oberkochen, Germany). All samples were coated by PVD-Sputtering with C-graphite before observation. Samples composition was analyzed with energy dispersive X-ray spectroscopy (EDX) [31,32].

The damaged surfaces (chipping pieces) were analyzed by grazing incidence X-ray diffraction (GI-XRD) using an X’Pert MPD automatic diffractometer (Philips Analytical, Eindhoven, the Netherlands) equipped with a thin film attachment and using Cu-Kα radiation. A range of 2θ = 20 until 50 ° was studied in continuous mode, under a fixed incidence angle of 0.5°. A step size of 0.02° was used in all scans, with a 2 s time step. In order to improve the signal-to-noise ratio, we also analyzed 5 samples in step mode with a time step of 10 s [33,34].

The roughness of the chipping species was determined by white light interferometry using a Wyko NT1100 Optical Interferometer (Veeco Instruments, USA) in vertical scanning interferometry mode with a vertical resolution of 2 nm. The analysis area was 112 × 90 µm. Data analysis was performed with Wyko Vision 32 software 4.20 update2 (Veeco Instruments, Plainview, New York, NY, USA), applying a Gaussian filter to separate waviness and form from roughness. The measurements were made in 3 different specimens of each type to characterize the amplitude parameter (Sa).

Fracture strengths were determined from different pieces of the balls fractured and from original balls. Five mechanical tests were realized in flexural method for each ball fractured. These tests were not in accordance with the standard because they involved fractured samples, however, the tests allowed us to view the trends of the mechanical behavior. The testing machine was a Bionix 858 MTS (Minneapolis, MN, USA) controlled by MTS Testworks 4 software.

Statistical analysis was performed using one-way ANOVA tables, Student’s *t*-tests, and Tukey’s multiple comparison tests. These analyses were carried out using Minitab^T^ 13.0 software (Minitab Inc., State College, PA, USA). The statistical differences were considered significant when *p* < 0.001.

## 3. Results

**Figure 2:**
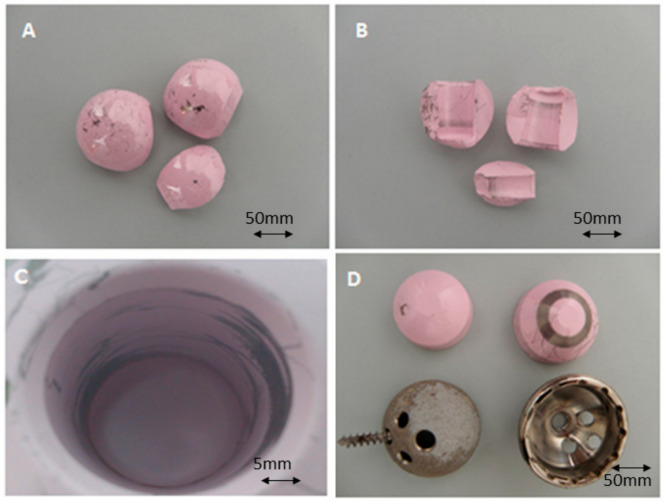
(**A**,**B**) Fractured head balls. (**C**) Smears appeared on the inner part of the bore. (**D**) The liner presented significant marks in the form of ring on the surface in contact with the metallic cup.

The received samples of the ceramic femoral head and ceramic insert were analyzed by FIB-SEM microscopy. The surface of the head ball as well as the bore presented heterogeneous dark smears throughout the samples (Figure 2A,B). The head bore, connecting the trunnion, presented a heterogeneous smear, and significant marks on the lower part of the bore close to the chamfer (Figure 2C). On the other hand, the insert presented circular ring-like smears on the surface (Figure 2D).

**Figure 3:**
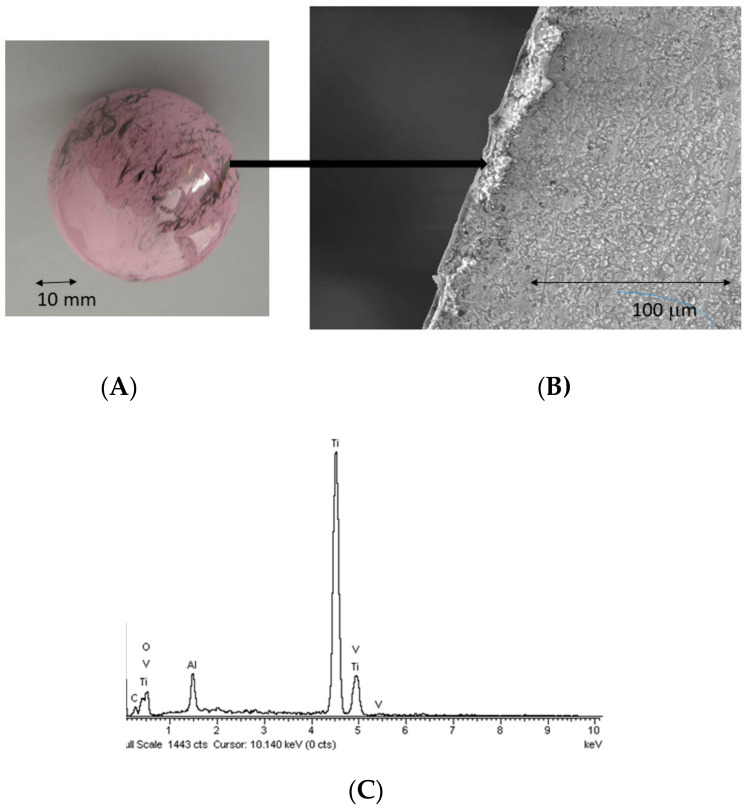
(**A**) Femoral head with metallic marks. (**B**) Section of ceramic observed by SEM with metal included. (**C**) SEM-energy dispersive X-ray spectroscopy (EDX) micronalysis spectra of the metal observed. The diffraction showed Ti-6Al-4V alloy.

Similar marks appeared on the opposite wall located in the upper part of the bore. SEM and EDX results indicated the presence of titanium on both smears (Figure 3). This pattern was indicative of an excessive wear on the indicated sites, probably indicative of an uneven fitting of the stem within the ball head. Other smears were observed in the surface of the ball, related with metallic debris that was transferred into the head, probably after the failure of the implant.

**Figure 4:**
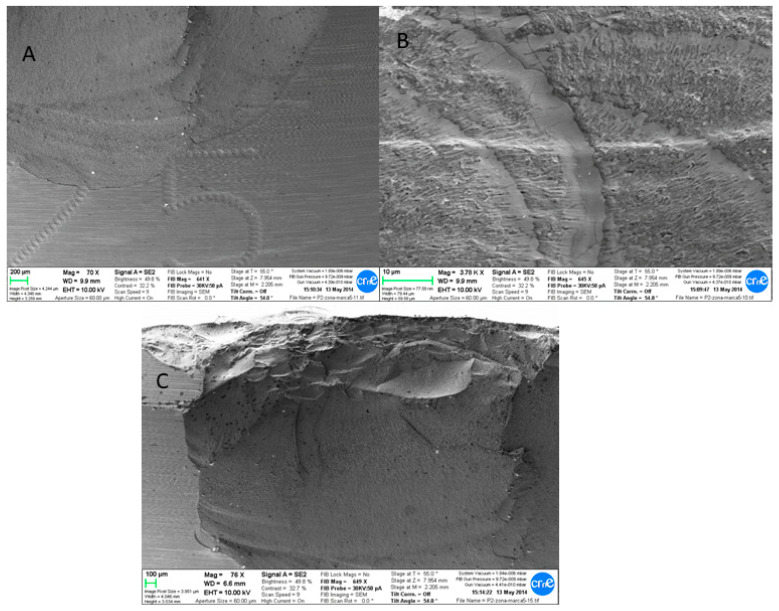
SEM fractographic images of fractured components: crack originated in the area surrounding the serial number (**A**). Cracks were present within the serial numbers (**B**). The propagation of the crack created a significant defect in the liner (**C**).

Their presence can also be clearly observed in the SEM images, showing titanium debris (Figure 4A,B). Figure 4B,C clearly shows the progression of cracks through where the head ball fractured. The parts that did not present smears did not present any titanium content either.

A group of representative SEM images of the fractured components are shown in Figure 4. Fractographic observations clearly showed a single-fracture behavior characterized by its brittle fracture type.

**Figure 5:**
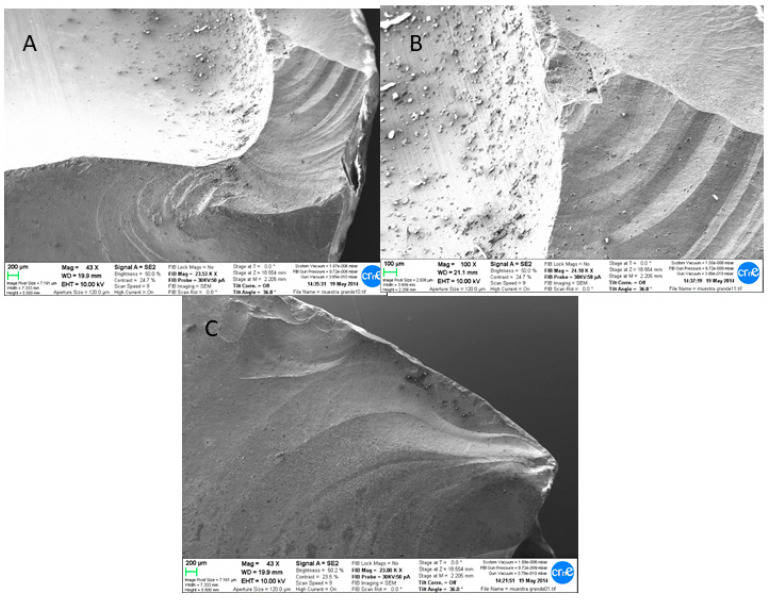
SEM fractographic images of the bore trunnion area clearly showed the crack formation and propagation, indicating the brittle fracture (**A**–**C**). The presence of titanium was present in the bore, as observed by the presence of debris.

Another finding was the presence of a crack initiator at the bottom of the insert, next to the serial number, indicating the possible stress concentrator, as shown in Figure 5. A group of representative SEM images of the fractured components are shown in Figure 5. Fractographic observations clearly showed a crack development surrounding the serial number as well as within the serial number.

No patient presented audible squeaking in any time.

The X-ray analysis of samples obtained in the retrieved ceramics can be observed in Table 1. This table summarizes the average monoclinic phase fractions measured on the surface of chipping species of femoral heads at different times. The patients were different and the conditions such as height and movement, among others, could modify the parameters. This was a limitation of the study. However, the analysis of the results presented in Table 1 shows both a progressive increase of the monoclinic phase as well as roughness with the time of implantation. The increment of monoclinic phase content with time of implantation would be associated with aging of the Y-TZP. In addition, roughness increase with time would be associated with volume changes related to the tetragonal to monoclinic phase transformation. The increase of the monoclinic phase produces an important decrease of the toughness and increase of the brittleness. Statistical analysis showed significance differences of the monoclinic phase percentage according to the time of implantation (*p* < 0.001). Moreover, the average surface roughness presented significance differences depending on the time of implantation (*p* < 0.001).

Table 1 also shows the values of the fracture resistance, observing how the maximum value corresponds to the original ball and how the degradation negatively affects the mechanical behavior of the zirconia. The values cannot be taken reliably as no established standard has been followed but it shows the clear trend of loss of mechanical properties.

## 4. Discussion

The appearance of ceramic-to-ceramic couples has shown great improvements in comparison with other couples such as ceramic–polymer, improving the wear friction and consequently reducing the number of particle debris arising from the wear [35,36,37]. Their unique properties have made ceramic–ceramic couples very promising couples for THA, showing a considerable evolution with time. Initially, the first generation ceramics had a component fracture rate of up to 0.026% [38]. These were then improved with the presence of the second generation of ceramic couples, which showed increased alumina purification and a decreased grain size. These changes were able to reduce the fractures up to only 0.014% [39]. The third generation ceramics, including Biolox, allowed the reduction of fractures up to 0.004% [39]. The main reason for this reduction in the fracture rates arises from the manufacturing process. Among several factors, the use of the most innovative manufacturing techniques such as HIP (hot isotactic pressing) and laser etching, as well as an exhaustive and monitored quality control, are probably the main reasons that would justify the aforementioned reduction in the fracture rates. The main benefit of the third generation of ceramics is that the crack propagation is hindered through the insertion of yttrium-stabilized tetragonal zirconia particles [40,41]. This stabilization was shown to be effective without an associated decrease in strength, roughness modification, or surface damage [42]. The combination of zirconia in the alumina matrix is able to allow only partial monoclinic transformation of the zirconia, since complete zirconia heads have been shown to have complete transformation of the zirconia and an increased risk of fracture, presenting over 340 fractures in a period of four years [43,44].

Despite the improvements of third generation ceramics, fractures still occur and need to be reduced in order to offer a commercial treatment without risks [45,46]. A group of different variables have been identified as increasing risk factors of CoC-bearing fractures, including ceramic composition [6,47], period of manufacture [45], small head size and short neck length adjustment [6,35], component design [41], manufacturing defects [6], thin liners [40,41,42], and high body weight [6]. Previous fractures exist with BIOLOX forte—among 359 implanted THAs, 5 patients presented head fracture. In these cases, a short neck hip was used and showed a fracture arising from a circular crack along the thin portion close to the bore [9]. Some other fractures were also observed that extended radially down to the lower edge of the component [42,47,48]. Up to the year 2000, BIOLOX Delta had not encountered any fractures after a 3-year follow up in 333 cases [42,47,48,49,50,51,52].

The most widely accepted hypothesis to explain this kind of metallic rim of acetabulum is related to the cyclic impingement between the neck of the stem and the acetabular component. The position of the stem, pelvis, and acetabular component plays an important role in this failure [6]. Moreover, the misalignment of the liner during impact loads into the acetabular component increases the risk of liner fractures [53,55,56,57].

In the present study, both the heads and the bore were marked with dark lines. The fracture of the head ball was aroused from the site of the peak tensile stress, which is located in the trunnion bore area. An impaired fitting of the trunnion and the head probably caused stresses in the taper head, producing the catastrophic failure. This catastrophic failure was a brittle fracture with rapid propagation of a vertical crack extended from the beginning of the bore to the lower edge of the chamfer [58,59,60,61,62]. Besides the improvement on the material design that has considerably reduced the rates of fractures, the design of the head is also very relevant in terms of reducing the rates of fractures. In this sense, the tapered hole in the femoral head is a key element in the design of femoral heads, where two main designs apply: the short neck tapers and the long neck tapers. In the short neck tapers, the contact between the bore of the head and the trunnion of the stem is the highest, presenting a short distance to the dome of the head, increasing the rate of fracture in the union between the top of the bore and the dome [63,64,65,66,67]. On the other hand, in the long neck tapers, the contact area is lowered between the bore of the head and the trunnion of the stem, presenting a long distance to the dome and having a high risk of failure in the lower part of the bore as a result of an increased stress at the superior rim of the bore [68,69,70,71,72].

Regarding the liner, dark metallic smears with circular morphology were observed on the back of each liner. The presence of titanium evidences constant scratching and wear due to the friction that occurs between the ceramic insert against the metallic surface of the cup. This process may be worsened if the screws are not securely seated in their spot seats and if some head protruding exists. Other possible causes of failure could be the incomplete sitting of the liner in the acetabular shell. It should be highlighted that titanium cups can deform during its implantation into the bone. Modifications of the titanium shell has been shown to seriously affect the fitting between them, increasing the chances of failure [73,74,75,76].

Furthermore, the liner presented another initial propagation site, which was allocated in the serial number region. Serial number is nowadays marked with laser as opposed to engraving, since it may reduce the cracks. In the present study, an excessive laser etching has been shown to be detrimental for the stability of the ceramic, acting as zones of higher energy through where cracks may start forming. Previous cases in which laser marking was applied on the neck–shoulder junction of the femoral stems has been shown to act as a stress-raiser, initiating a fatigue fracture [77,78,79,80]. Despite the different nature of the material, the principle can be taken as very similar, since the increase in stress in the microenvironments surrounding the number could have acted as stress concentrator in the ceramic.

The damage produced in the surface of the zirconia heads is due to the presence of zones that have an important percentage of monoclinic phase, as can be proved by X-ray diffraction (Table 1). This phase possesses a low toughness with risk of fracture in service. The presence of yttria has not been detected in these particles, which would stabilize the tetragonal phase, being the cause of the increase of the fracture toughness of the zirconia. This degradation of the yttria-tetragonal stabilized zirconia to monoclinic phase, which presents low mechanical properties, is due to the exposition to humid environments at body temperature, which is well known as a hydrothermal ageing phenomenon (low temperature degradation). This degradation favors the presence of alumina. Several hypotheses have been proposed about the interaction of the alumina with the water, but the theories have not yet been truly understood [78,79,80,81,82,83]. Some aspects are well known, such as the fact that the degradation starts from the surface and proceeds to the ceramic bulk inwards. The solid transformation from tetragonal to monoclinic phase is accompanied with an increase of volume, producing an increase of roughness or even microcracks, voids, and uplifts, and, as a consequence, chipping in the ceramic. The zirconia femoral head exhibits a dramatic increase of roughness from 4 nm to more than 50 nm. This fact was observed by other authors [60,74]. As a consequence of this increase of roughness, an acceleration of the wear rate of the hip component is observed and may cause clinical revision by metallosis disease. Titanium alloys present poor values of wear resistance due to their lower hardness compared to ceramic materials. In terms of reducing the wear in the Morse cone of the hip prosthesis, Cr–Co–Mo alloy is better than titanium alloys. Co–Cr alloys present higher hardness and better wear resistance than titanium alloys [48,49,81,82,83,84,85,86]. Chevalier et al. [73] demonstrated that the tetragonal-to-monoclinic transformation can propagate from one grain to the adjacent grains, giving a large growth rate. In the case of zirconia–alumina composite, the alumina grains surrounding each zirconia grain hinder the transformation propagation to the neighbors [87].

The incorporation of alumina in the ceramic increases the hardness; this oxide presents a hardness around 2000 Hardness Vickers Number harder than zirconia and more than 10 harder times compared with Ti-6Al-4V. This hardness provides improved scratch resistance, but the wear against Ti-6Al-V is produced easier, as can be observed in the Morse cone.

This study presents some limitations regarding of any variables not controlled for that differ among a human cohort such as age, movement and physical activity, height and patient anatomy, or weight. However, a high number of cases has been reviewed to obtain consistent results. It has been observed that sex is not critically important since the failures have been given in a similar number for men and women. It is worth highlighting that the number of incidents was small and did not allow for any type of statistical analysis or any definitive conclusion. However, the idea of collecting more abundant series, other THA types, or greater number of removed implants is ongoing in order to reach further conclusions.

Lastly, the novelty of this study is its analysis of the early mechanical failures that can be observed in CoC THAs of the last generation in young patients. The results showed that total safety after receiving a THA is still a goal to be pursued. In spite of having more than 100 patients and having an almost 5% failure rate (high percentage), there are many parameters that affect the mechanical behavior due to the differences between the patients. However, this work provides conclusions about the long-term behavior of the studied hip prosthesis. It would be convenient to study more cases, although, as we have seen, different authors show similar conclusions.

## 5. Conclusions

The present study shows the need to further improve the third generation of ceramic-on-ceramic-bearing couples for total hip arthroplasty. Despite the improved wear compared to other materials, stress concentrators are sources of initial crack propagation, such as those found in the bore trunnion areas. The presence of these cracks is considered as the previous step of the brittle fractures observed. Reducing the stress concentrator needs to be strongly considered in order to improve their efficiency and reduce the risk of catastrophic brittle fractures. Moreover, in light of the evidence observed in this study, the chipping observed was due to the presence of monoclinic phase of the Y-TZP instead of tetragonal, which presents better mechanical properties. Alumina and the presence of water produced a low temperature degradation of the Y-TZP, producing brittle fractures of the monoclinic particles.

## Figures and Tables

**Table 1:** Analysis of the chipping ceramics of four fractured prostheses with different time of implantation. Monoclinic phase content and roughness are analyzed. The standard deviation is found within the parentheses.

Prosthesis Reference	Time of Implantation (Days)	Monoclinic Phase (%)	Roughness (nm)	Fracture Resistance (N)
Original	0	14 (5)	4 (2)	978 (67)
47-FJM	372	25 (7)	11 (4)	934 (30)
92-XGM	398	38 (9)	19 (3)	925 (11)
98-MSO	410	42 (9)	19 (7)	910 (38)
103-REQ	500	45 (7)	21 (5)	901 (21)
108-SAT	587	50 (13)	32 (3)	889 (30)
110-TAG	639	75 (11)	55(4)	860 (40)

## Supplementary Materials

The following are available online at https://www.mdpi.com/1996-1944/13/23/5318/s1, Table S1: List of the 117 patients: age, sex, hip prosthesis placement, revisions.
